# Study on the Preparation and Properties of Impervious and Breathable Sand Based on Hydrophobic Theory

**DOI:** 10.3390/ma14195613

**Published:** 2021-09-27

**Authors:** Xiao Li, Xiong Zhang, Hao Ren

**Affiliations:** 1 Key Laboratory of Advanced Civil Engineering Materials of Ministry of Education, Tongji University, Shanghai 201804, China; 1932894@tongji.edu.cn (X.L.); 1830691@tongji.edu.cn (H.R.); 2 School of Materials Science and Engineering, Tongji University, Shanghai 201804, China

**Keywords:** impervious, breathable, hydrophobic, surface rough structure, coating materials

## Abstract

Land desertification, a severe global ecological and environmental problem, brings challenges to the sustainable utilization of land resources in the world. The purpose of this research is to use hydrophobic theory to prepare impervious and breathable sand, and to solve the problems of sandy soil that seeps easily and makes it difficult for vegetation to survive in desertified areas. The influences of coating material content, first-level and second-level rough structure on the impermeability and air permeability of impervious and breathable sand were studied. The research showed that, with the increase in coating material content, the impervious performance of the sample increased firstly and then decreased, and the air permeability rose continuously. The hydrostatic pressure resistance of the sample can reach an extreme value of 53 mm. The first-level rough structure of micron structure can greatly improve the hydrophobic performance, thus improving the impervious performance. The addition of micron calcium carbonate would improve the hydrostatic pressure resistance height of the sample to 190 mm. The sample would reach a superhydrophobic state in the condition of a first-level rough structure of a nano structure built by nano silica, and the contact angle was up to 152.0°, so that the hydrostatic pressure resistance height can rise to 205 mm. The best performance would be achieved under the condition of relatively less raw material with a second-level rough structure of micro–nano. At this point, the contact angle of the sample reached 152.8° and the hydrostatic pressure resistance height was up to 205 mm. At the same time, the air permeability index of the above four kinds of impervious and breathable sand met all planting requirements. The sample prepared can satisfy the demands of different degrees of impermeability and air permeability, and can be widely used in desertification control.

## 1. Introduction

Currently, one of the most severe ecological problems facing the world is land desertification [1,2]. Land desertification will make the soil severely sanded, meaning that the water retention of the soil will drop sharply [3,4]. This makes it difficult to provide sufficient water and organic matter for vegetation [5]. In order to solve the problem of vegetation growth in land desertification areas, many scholars have conducted research on impervious and breathable materials. Under the premise of preventing water leakage, this type of material can also ensure the air permeability of the material used to promote the root absorption of plants [6], which brings new possibilities for vegetation growth in desertified areas.

Many researchers have conducted related studies on impervious materials. Siddiqui et al. [7] studied artificial impervious surfaces of HDPE (High Density Polyethylene) on the urban ground, but environmental stress would be caused by the large amount of HDPE used. D. Barnate-Hunek et al. [8] used polystyrene modified concrete for hydrophobic modification of silicone compounds. However, during the preparation of the concrete, a large amount of carbon dioxide was generated [9], and its freeze–thaw resistance ability under extreme weather would be reduced [10]. In addition, these impermeable materials were not up to the standard of plant breathability. Based on this, some scholars have proposed to use cheap and easily available sand as a base material to prepare the hydrophobic system by using its natural breathable advantage, so that the sand particles have both impervious and breathable properties to achieve the purpose of “controlling desert with sand”. Men X.H. et al. [11] used octadecyltrichlorosilane to hydrophobically modify sand. Due to the extremely fine particle size of the sand used, it exhibited superhydrophobic properties, but the scholars mainly applied it in the field of oil–water separation. Chen L.W. et al. [12] used chemical methods to construct three different nano-rough structures of silica, metallic silver, and metallic copper on the surface of sand particles with a low surface energy material, to obtain three types of superhydrophobic sands. These superhydrophobic sands can maintain good performance in extreme environments, bringing new hope for desert water storage and transportation. At the same time, the researcher [13] also used copper hydroxide nano-needles to coarse the surface of sand particles, thus a new type of superhydrophobic sand was obtained and applied to the oil–water separation field. Other scholars, such as Liu J.L. [14] and Liu P.S. [15], also modified sand with hydrophobicity, and applied it in the field of oil–water separation. Luo T.X. et al. [16] used phenolic resin to coat the surface of sand particles to form a hydrophobic layer, to give the modified sand particles a certain hydrophobicity, and applied it in the field of waterproofing. Zhang Z.Z. et al. [17] modified the surface of soil particles with wood wax, and sprayed the soil particles on the sand surface to form a breathable and water-inhibiting film, but the film could be deteriorated when the sand particles were moved. Therefore, we can find from the existing research that there are many methods to prepare hydrophobic sand particles. However, there are still some problems in current research: regarding the chemical methods that are predominantly used to prepare impervious and breathable sand, their preparation process make it difficult to achieve industrial production, and there were few studies on the application of hydrophobic sand to desertification control. Therefore, it is urgent to prepare a kind of impervious and breathable sand with a simple preparation method and excellent performance, to study its effect in the field of desertification treatment.

Based on this, this paper intended to develop a convenient preparation process to make impervious and breathable sand. The previous study shows that there are two factors affecting the wettability of material surface [18,19]: one is surface energy, the other is surface roughness. Therefore, sand particles were used as the core material, and the mixture of fluorocarbon resin with low surface energy and isocyanate curing agent were applied to cover the sand particles, and to evaluate the influence of coating material on the performance of impervious and breathable. On the basis of film coating, micron, nano and multi micro–nano coarsening methods were carried out successively to study the influence of surface roughness structure [20,21,22] on the performance of impervious and breathable.

## 2. Test Materials and Methods

### 2.1. Materials

The raw sand was purchased from Fuxin Rituo Silicon Sand Co., Ltd. (Zhangwu County, Fuxin City, Liaoning Province, China) and its particle size distribution is shown in Table 1. The coating materials include fluorocarbon resin (referred to as FC) and isocyanate curing agent (mixed at 10:1), obtained from Shanghai Fluorkang Co., Ltd (Jinshanwei Town, Jinshan District, Shanghai, China). The coarsening materials, including hydrophobic micron calcium carbonate (2–10 μm) and hydrophobic nano silica (20–70 nm), were the chemical reagents.

### 2.2. Sample Preparation

**Figure 1:**
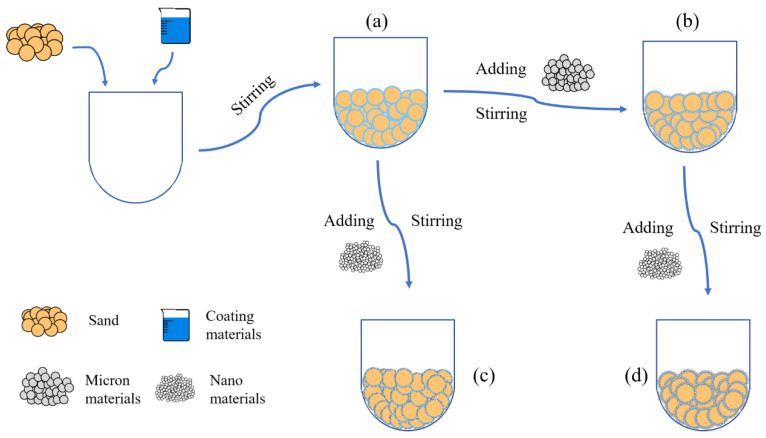
Flow chart of sample preparation: (**a**) Pure-coated sample; (**b**) Pure micron type; (**c**) Pure nano type; (**d**) Micron-nano type.

According to the surface structure of the material, the impervious and breathable materials can be divided into four categories: pure-coated type, pure micron type, pure nano type and micro–nano type. From Figure 1, the specific preparation process can be summarized as follows: The pure-coated sample can be obtained by adding 1–4% coating material into the sand and stirring at the speed of 140 r/min evenly. On this basis, pure micron or pure nano samples can be obtained by adding 0.5–2.5% micron materials or 0.1–0.8% nano materials. Similarly, micro–nano samples can be obtained by adding 0.1–0.3% of nano materials to a pure micron sample with a micron material content of 1%.

### 2.3. Test Methods

The test methods adopted in this paper were hydrostatic pressure resistance height test, air permeability index test, contact angle test, SEM test and particle size analysis test. Among them, SEM, contact angle and hydrostatic pressure resistance height tests were mainly used to characterize the hydrophobic and impervious properties of the materials. When the liquid droplets contacted with the solid surface, they would penetrate into the grooves on the material surface. Therefore, if the material surface had enough roughness, the contact area between the liquid droplets and the solid surface would be increased, so that the water droplets needed more energy to infiltrate the surface. This further improved the impermeability of the material. The air permeability index and particle size analysis test were mainly used to test and explain the permeability of samples.

**Figure 2:**
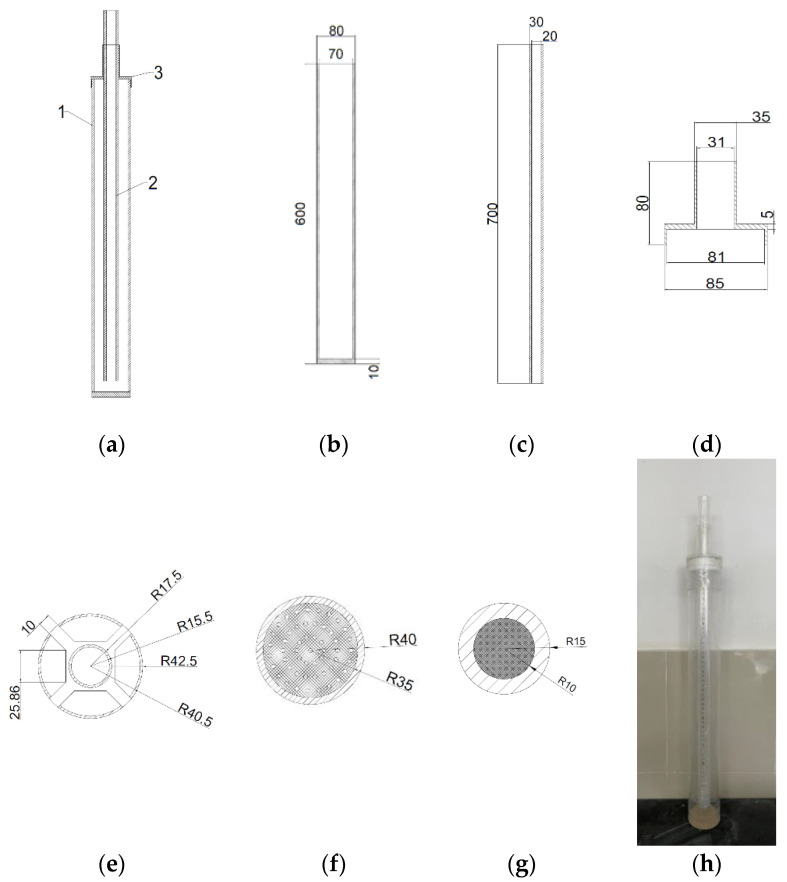
Schematic and physical diagram of the hydrostatic pressure resistance height measuring instrument: (**a**) Overall main (side) view; (**b**,**f**) The main (side) view and top view of outer cylinder respectively; (**c**,**g**) The main (side) view and the top view of inner cylinders respectively; (**d**,**e**) The main (side) review and top review of the center fixing frame; (**h**) The physical diagram of the hydrostatic pressure resistance height measuring instrument. Note: The dimensions shown were all in millimeters.

The impervious performance was characterized by a hydrostatic pressure resistance height test by using a self-made instrument. From Figure 2, the device was mainly divided into 3 parts; 1 represented the outer cylinder, 2 represented the inner cylinder, 3 represented the center fixing frame. In addition, the bottom of the outer cylinder should be drilled and sealed with a fine screen to avoid sand particles leaking from the hole, and the inner cylinder should be sealed with a fine iron sheet. Firstly, a 2 cm thick sample was poured into the outer cylinder. Secondly, the inner cylinder was inserted into the outer cylinder and an appropriate amount of sample was loaded into the gap between them. Finally, water was added into the inner cylinder at a certain speed and it was observed whether there was water leakage in the bottom of the outer cylinder. In the case of water leakage, the height of water column in the inner cylinder was taken, that is, the height of hydrostatic pressure of the sample. Each sample was tested 3 times, and the average value was taken as the hydrostatic pressure resistance height value, to a precision of 1 mm.

Air permeability of materials was assessed by a direct reading from a breathable instrument, according to the standard GB/T 2684–2009 “Test method for sand and mixture for foundry” [23]. Firstly, a 5 cm high sample was added to the sample barrel and compacted. Then, it was put into the sample table and the instrument was adjusted to the working preparation state. Each sample was measured 3 times and the average value was taken, the accuracy was 1 cm^2^·Pa^−1^ min^−^^1^.

The hydrophobic performance was characterized by a contact angle tester DSA25 purchased from Kruss Scientific Instruments (Shanghai) Co., Ltd. (Chundong Road, Minhang District, Shanghai, China). The following steps were taken. Firstly, water was taken as a test drop, and then the camera system of the instrument was used to photograph the shape of the drop on the sample surface, and the contact angle value was recorded. Three different areas of the sample were selected for testing, and the average was taken as the result, to a precision of 0.1°.

The surface morphology of sand particles was characterized by a Schottky field emission scanning electron microscope. Under the conditions of acceleration voltage 10 kV and spot size 3.0, the annular backscattered electron images of the sample surface at different scales were collected.

Particle size analysis was carried out by a screening test referring to JC 52-2006 “Standard for Quality and Inspection Methods of Sand and Stone for Ordinary Concrete” [24]. The samples were screened with a mesh size of 0.6 mm, 0.5 mm, 0.4 mm, 0.3 mm, 0.2 mm and 0.1 mm, respectively. Firstly, a 500 g sample was weighed and screened according to the standard. Then, the quality of the samples on each screen was weighed, and the sieve residue of each layer was calculated, accurate to 0.1%.

## 3. Results and Discussion

### 3.1. The Influence of the Coating Materials

**Figure 3:**
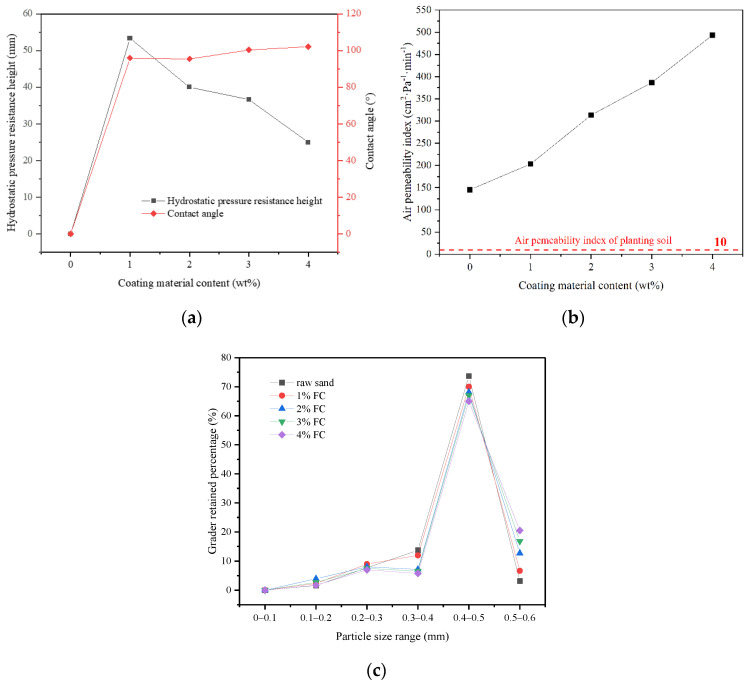
Influence of the coating material on the properties: (**a**) The influence of coating material content on the hydrostatic pressure resistance height and contact angle; (**b**) The influence of coating material content on the air permeability index; (**c**) The grader retained percentage of materials with different coating material content.

**Figure 4:**
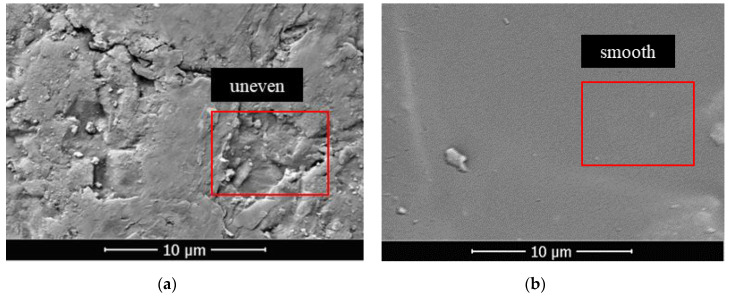
(**a**) SEM images of sand particle surface before film coating; (**b**) SEM images of sand particle surface after film coating.

**Figure 5:**
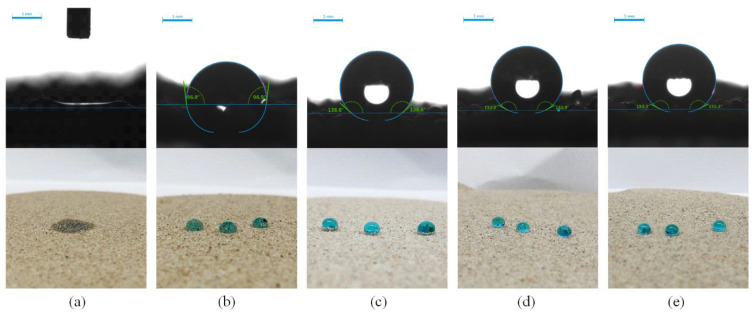
The water contact angle of the test diagram and real diagram of raw sand and different types of samples: (**a**) Test diagram and real diagram of raw sand; (**b**) Test diagram and real diagram of pure-coated type; (**c**) Test diagram and real diagram of pure micron type; (**d**) Test diagram and real diagram of pure nano type; (**e**) Test diagram and real diagram of micro–nano type.

In this section, FC was used as a low surface energy material to prepare pure-coated samples. From Figure 3a, the impervious performance of the sample showed a trend of rising firstly, and then failing with the increase in coating material content: When there was no film coating, the hydrostatic pressure resistance height of the sample was 0 mm. When 1% FC was applied, the hydrostatic pressure resistance height of the sample reached the maximum value, 53 mm. When 4% FC was coated, the hydrostatic pressure resistance of the material dropped to 25 mm. It was indicated that the sample would show impermeable performance with an appropriate amount of coating materials [25]. Because of the low polarizability of the fluorine atom, the polarity of fluorocarbon resin molecules became smaller. Therefore, the surface-free energy was reduced, due to the smaller intermolecular force, giving it a certain hydrophobicity. At the same time, due to the large electronegativity of fluorine atoms, when water molecules contacted with resin, the cohesion between water molecules was much greater than the attraction between water molecules and fluorine atoms. This made the contact angle of the sample to water increase [26,27]. From Figure 4a,b, the surface of the raw sand was uneven, but after film coating, the surface became smooth and even, implying that the incorporation of FC formed a hydrophobic layer on the surface of the sand particles. From Figure 5a,b, compared with the raw sand, the pure coated sample showed hydrophobic properties. However, the more the coating material was added, the more serious the group gathering between particles. Therefore, the impervious performance decreased due to the increase in the average particle size. From Figure 3c, the particle size distribution of the sample coated with 1% FC was basically the same as the original sample. However, with the increase in coating material content, the particle weight in the range of 0.3–0.4 mm decreased significantly, and the particle weight in the range of 0.5–0.6 mm increased remarkably. The larger the coating material content, the more significant the change of particle weight, resulting in the increase average particle size, which proved to be the reason that the hydrostatic pressure resistance height changed with the coating material content.

It can also be seen from Figure 3a that the contact angle of the sample increased from 96.9° to 102.1° with the increase in coating material content (more than 1%). The small increase can be attributed to the same hydrophobicity of the material, indicating that the hydrophobicity of the sample was not affected by coating material content when the content was sufficient. In addition, from Figure 3b, the air permeability index of all the samples was greater than the air permeability requirement of the planting soil at 10 cm^2^·Pa^−1^ min^−1^ [28]. The air permeability index increased from 145 cm^2^·Pa^−1^ min^−1^ to 493 cm^2^·Pa^−1^ min^−1^ with the increase in coating material content. The increase in coating material content leaded to the particle agglomeration, the increase in the average particle size of particle group and the rise of the space between particles, so as to improve the air permeability, which can also be demonstrated by the particle size analysis test results. Therefore, the pure-coated samples were equipped with a certain degree of hydrophobicity, and met the planting requirements of air permeability, which could alleviate the problem of land desertification to a certain extent.

### 3.2. The Influence of Surface Rough Structure

As mentioned above, another way to prepare a superhydrophobic surface is to construct rough surface structures. Some scholars have found that micro–nano rough structure can directly construct superhydrophobic surfaces, but this is not the only way. We took inspiration from nature, where many surfaces with superhydrophobic properties were equipped with first-level rough structures [29] (such as cicadas [30]) or second-level rough structures (such as lotus leaves and water striders [31]). It is indicated that we can improve the superhydrophobic properties by constructing an appropriate microscopic morphology on the surface. Therefore, in this section, the influence of surface roughness on the performance of prepared products was investigated by setting up a first-level rough structure (pure micron type or pure nano type) and a second-level rough structure (micro–nano type).

#### 3.2.1. First-Level Rough Structure

**Figure 6:**
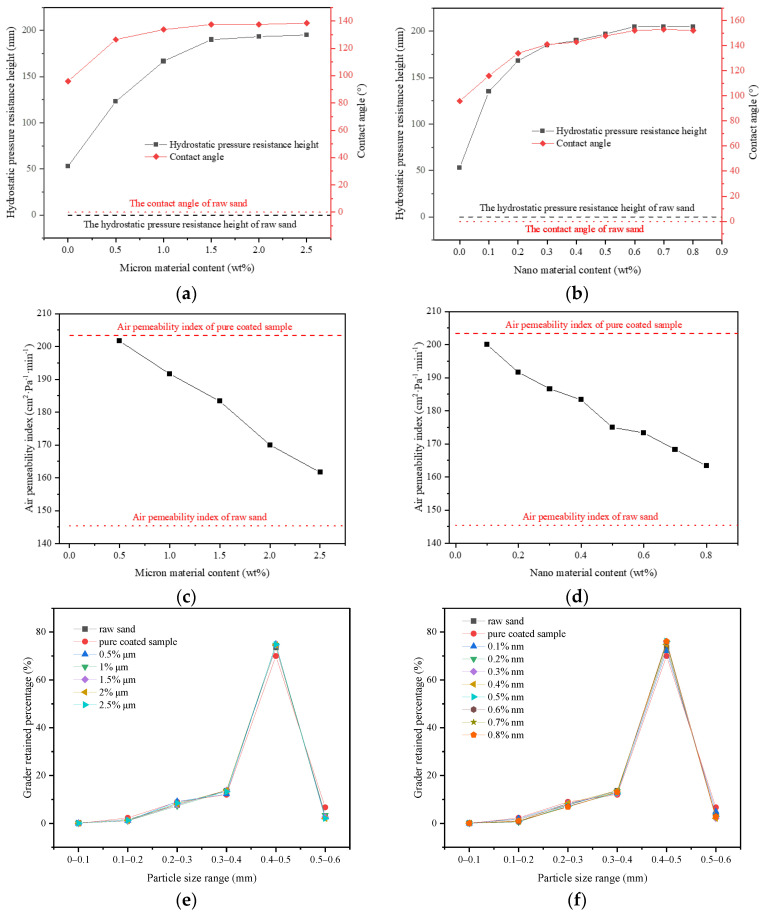
Influence of the rough structure of pure micron type and pure nano type on properties: (**a**,**b**): Effect of pure micron type and pure nano type structure on hydrostatic pressure resistance height and contact angle of materials; (**c**,**d**): Effect of pure micron type and pure nano type structure on the air permeability index of materials; (**e**,**f**): The grader-retained percentage of materials with different micron or nanometer content.

**Figure 7:**
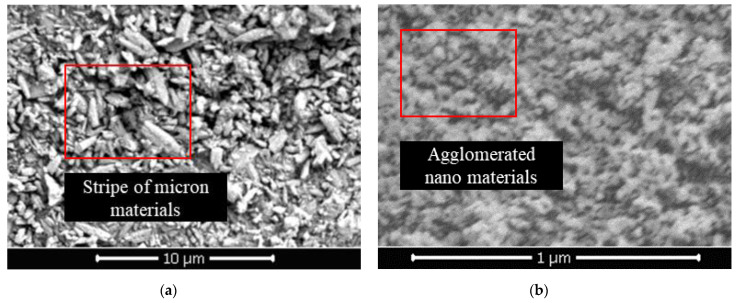
(**a**) SEM image of pure micron type; (**b**) SEM image of pure nano type.

In this section, 1% coating material was used as the benchmark to prepare the pure micron level or pure nano level samples by changing the content of micron material or nano material, and to explore the influence of first-level structure on properties. From Figure 6a,b, both the hydrostatic pressure resistance height and contact angle of the sample firstly increased and then tended to be stable. When micron material content was in the range of 0–1.5%, the hydrostatic pressure resistance height of the pure micron type sample rose from 53 mm to 190 mm, increasing by about 258%. The contact angle rose from 96.9° to 137.5°, which suggested that the addition of micron material significantly improved the hydrophobic performance. According to the Wenzel and Cassie theoretical model [32,33], when the intrinsic contact angle of the material was greater than 90°, the increase in roughness would lead to the improvement of hydrophobicity. From Figure 7a, the strip micron materials were adhered to the surface of the pure-coated sample to form a micron-scale rough structure, which improved the surface roughness and greatly enhanced the hydrophobic performance of the sample (Figure 5c), contributing to the boost of impervious performance [34]. When the micron material content reached 1.5%, the hydrostatic pressure resistance height and contact angle of the sample basically tended to be stable, and reached the extreme value, illustrating that the content of 1.5% micron material had been saturated.

From Figure 6b, when the content of nano material was in the range of 0–0.6%, the hydrostatic pressure resistance height of pure nano type sample increased from 53 mm to 205 mm, increasing by about 287%. The contact angle increased from 96.9° to 152.0°, reaching the superhydrophobic state (contact angle >150° [35,36]). This manifested that the addition of nano material remarkably improved the hydrophobic performance, and even made the sample present superhydrophobic properties (Figure 5d). A large number of agglomerated nano particles stuck to the surface of pure-coated samples, forming a nano-scale rough structure (Figure 7b) to improve the surface roughness. Therefore, the hydrophobic performance of the samples was greatly strengthened, and the impervious performance was significantly enhanced. Similar to the pure micron type, the content of 0.6% nano material was saturated. 

In addition, from Figure 6c,d, when the micron material content was 0–2.5%, the air permeability index of the pure micron sample declined from 203 cm^2^·Pa^−1^·min^−1^ to 161 cm^2^·Pa^−1^ min^−1^. When the content of nano material was 0–0.8%, the air permeability index of pure nano samples dropped from 203 cm^2^·Pa^−1^ min^−1^ to 163 cm^2^·Pa^−1^ min^−1^, that is, with the increase in the content of micron materials or nano materials, the air permeability index of the samples decreased gradually and approached the air permeability index of the raw sand (145 cm^2^·Pa^−1^ min^−1^). The micron or nano material adhered to the coating layer on the surface of the pure-coated sample, forming the micron or nano rough structure (Figure 7a,b). At this point, the exposed area of the coated layer on the surface decreased, so that the agglomeration phenomenon between particles was weakened. The particle size distribution of the sample gradually tended towards the particle size distribution of the raw sand (Figure 6e,f). Therefore, the air permeability index of the sample decreased gradually with the increase in the content of micron material or nano material and tended towards the air permeability index of raw sand.

#### 3.2.2. Second-Level Rough Structure

**Figure 8:**
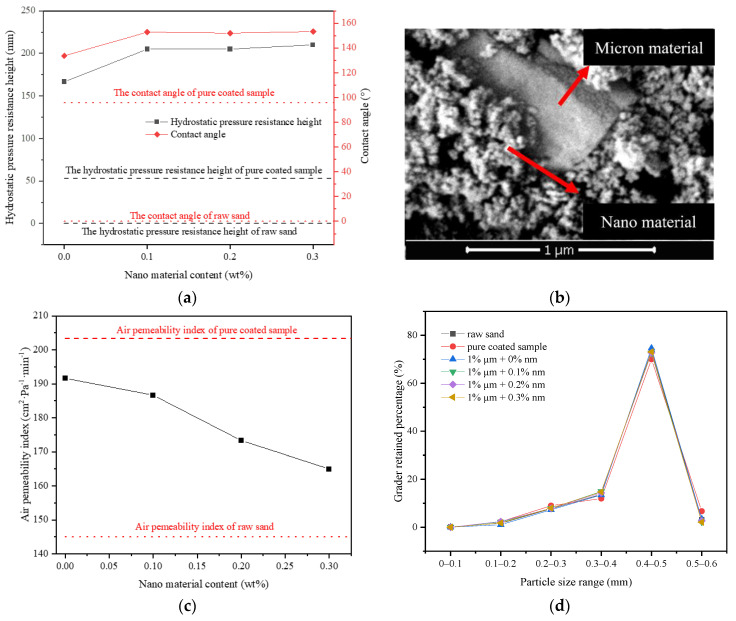
Influence of micro–nano type surface rough structure on properties: (**a**) Influence of micro–nano structure on hydrostatic pressure resistance height of materials; (**b**) SEM image of micro–nano type; (**c**) Influence of micro–nano type structure on the air permeability index of materials; (**d**) The grader-retained percentage of materials with different micron and nanometer content.

According to the above test results, 1% FC film coating and 1% micron material were taken as the benchmark to prepare multi micro–nano scale roughness surface sample by changing the content of nano materials, and to explore the influence of the second-level rough structure. From Figure 8a, both the hydrostatic pressure resistance height and contact angle of the sample firstly rose and then reached a maximum value. When the nano material content was 0–0.1%, the hydrostatic pressure resistance height of the sample increased from 167 mm to 205 mm, which was a boost of 23%. The contact angle increased from 133.7° to 152.8°, showing the superhydrophobic state (Figure 5e). From Figure 8b, a large number of nano particles were attached to the surface of raised micron particles, which constructed the micron-nano rough structure on the surface of the sample [37]. The rough structure greatly improved the hydrophobic performance of the material to a superhydrophobic condition, thus improving the impermeability of the sample. When the nano material content reached 0.1%, the hydrostatic pressure resistance height and contact angle of the sample remained basically unchanged and reached the extreme value, implying that under this structure, the nano content of 0.1% was saturated.

From Figure 8c, when the content of nano material was 0–0.3%, the air permeability index of the sample dropped from 192 cm^2^ Pa^−1^·min^−1^ to 165 cm^2^ Pa^−1^·min^−1^, that is, by raising the nano material content, the air permeability index of the sample declined gradually, and approached the air permeability index of raw sand. This was also because the incorporation of micron and nano materials weakened the agglomeration phenomenon among particles, making the particle size distribution of particle groups gradually closer to that of raw sand (Figure 8d), thus reducing the size of interparticle voids, which lead to a reduction in the air permeability index. 

Therefore, the performance of samples of first-level and second-level rough structures were greatly improved. Even the samples with first-level of nano rough structure and with second-level rough structure can achieve a superhydrophobic state. At the same time, the prepared sample can also take into account the air permeability, making it ideal material for desertification control.

### 3.3. The Mechanism of Impervious and Breathable Sand

**Figure 9:**
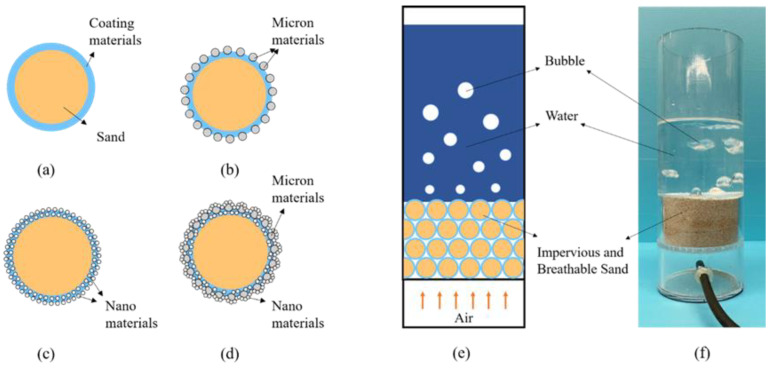
Four types of impervious and breathable sand and the mechanism of impervious and breathable sand: (**a**) Pure-coated type; (**b**) Pure micron type; (**c**) Pure nano type; (**d**) Micro–nano type; (**e**) The simulation diagram of the mechanism of impervious and breathable sand; (**f**) The physical form of the mechanism of impervious and breathable sand.

Impervious and breathable sand with four structures can be summarized by the methods mentioned above (Figure 9a–d). The impervious and breathable mechanism was shown in Figure 9e,f. The impervious and breathable sand accumulated into layers, with the water layer above and the air layer below. When the sand particles possessed a certain hydrophobic performance and were piled up together, they would obtain hydrostatic pressure resistance height, that is, impervious performance. From Figure 9a, the coating material with low surface energy formed a hydrophobic layer on the surface of particle sand, giving the sand hydrophobic and impervious performance. From Figure 9b–d, micron materials and nano materials constructed a first-level or a second-level rough structure on the surface of coated samples. This greatly improved the hydrophobicity of the sample, so that the water holding height of the sand layer increased, namely, the impervious performance was enhanced. 

In addition, due to the natural permeability of sand particles, air can pass upward through the void of sand particles easily, which satisfies the breathable needs of plant growth. Therefore, the four kinds of samples prepared in this paper were equipped with both impervious and breathable performance.

## 4. Conclusions

(1) The sand particles would be provided with hydrophobic properties with the addition of coating materials. With the increase in coating material content, the impervious performance increased, firstly to the extreme value of 53 mm, and then decreased, and the air permeability index showed a gradual rising trend, which met the requirement of vegetation growth.
(2) Using micron or nano materials to construct first-level rough structures on the surface of coated samples can greatly improve the hydrophobic performance. It can then enhance the impervious performance. This was able to reach 190 mm and 205 mm, respectively. The first-level rough structure of nano materials can even make the samples reach superhydrophobic state. The addition of micron or nano materials weakened the agglomeration phenomenon, and the air permeability index of the sample decreased gradually, but it still met the demand of permeability.
(3) Using micron and nano material to build second-level rough structures on the surface of coated samples can also greatly improve the hydrophobic performance of sand particles to reach the superhydrophobic state. This improves the impervious performance to the maximum of 205 mm. The permeability of the samples prepared by this method can also satisfy the requirement of planting. Furthermore, the micro–nano type made the sample performance reach its best under the condition of a minimum relative amount of raw material.
(4) In this paper, a simple and effective method was used to hydrophobically modify cheap and easily available sand, using a convenient process and giving it excellent performance. At the same time, the prepared samples can meet different impermeability and air permeability requirements, thereby alleviating the problem of easy leakage of water in desertified areas. This brings new prospects to desertification control.

## Figures and Tables

**Table 1:** Particle size distribution of raw sand.

Particle Size	0–0.1 mm	0.1–0.2 mm	0.2–0.3 mm	0.3–0.4 mm	0.4–0.5 mm	0.5–0.6 mm
Sieve residue	0.0%	1.6%	7.7%	13.7%	73.7%	3.2%
